# CYP24A1 Exacerbated Activity during Diabetes Contributes to Kidney Tubular Apoptosis via Caspase-3 Increased Expression and Activation

**DOI:** 10.1371/journal.pone.0048652

**Published:** 2012-10-31

**Authors:** Alexandre Tourigny, Frédrick Charbonneau, Paul Xing, Rania Boukrab, Guy Rousseau, René St-Arnaud, Marie-Luise Brezniceanu

**Affiliations:** 1 Université de Montréal, Centre de recherche de l'HSCM, Hôpital du Sacré-Coeur de Montréal, Montreal, Quebec, Canada; 2 McGill University, Shriners Hospitals for Children, Montreal, Quebec, Canada; University of Tokushima, Japan

## Abstract

Decreases in circulating 25,hydroxyl-vitamin D3 (25 OH D3) and 1,25,dihydroxyl-vitamin D3 (1,25 (OH)2 D3) have been extensively documented in patients with type 2 diabetes. Nevertheless, the molecular reasons behind this drop, and whether it is a cause or an effect of disease progression is still poorly understood. With the skin and the liver, the kidney is one of the most important sites for vitamin D metabolism. Previous studies have also shown that CYP24A1 (an enzyme implicated in vitamin D metabolism), might play an important role in furthering the progression of kidney lesions during diabetic nephropathy. In this study we show a link between CYP24A1 increase and senescence followed by apoptosis induction in the renal proximal tubules of diabetic kidneys. We show that CYP24A1 expression was increased during diabetic nephropathy progression. This increase derived from protein kinase C activation and increased H_2_O_2_ cellular production. CYP24A1 increase had a major impact on cellular phenotype, by pushing cells into senescence, and later into apoptosis. Our data suggest that control of CYP24A1 increase during diabetes has a beneficial effect on senescence induction and caspase-3 increased expression. We concluded that diabetes induces an increase in CYP24A1 expression, destabilizing vitamin D metabolism in the renal proximal tubules, leading to cellular instability and apoptosis, and thereby accelerating tubular injury progression during diabetic nephropathy.

## Introduction

Dramatic increases in the prevalence of diabetes and obesity rank them now among the most common and costly health problems encountered in developed countries [1], [2], [3]. Prevalence of type 2 diabetes has also increased markedly in recent decades, not only amongst adults, but amongst children as well [4], [5], [6]. Major clinical and scientific studies were conducted in the past years, trying to investigate and understand the role of vitamin D altered metabolism during type 2 diabetes. Nevertheless, we are far from a complete understanding of the mechanistic implications of vitamin D metabolism in disease progression. Lately, major breakthroughs have been done by investigating the role of CYP24A1 in chronic kidney disease progression [7], [8]. While data from clinical trials tell us of the importance of 25,hydroxyl-vitamin D3 (25 OH D3) and 1,25 dihydroxyl-vitamin D3 (1,25 (OH)2 D3) imbalance in diabetic patients [9], [10], most mechanistic studies have been conducted in cancer or genetically immortalized cells. These cells have altered signaling mechanisms that allow them to survive in hostile environments. Few studies were conducted in primary cells or freshly isolated cellular structures, and again only a handful of mechanistic studies were done in animal models [7], [11]. This study aims to better understand the role and mechanism of vitamin D metabolism in renal proximal tubules, during diabetic nephropathy progression.

Data from literature and our own preliminary work suggest that impaired vitamin D metabolism may be implicated in apoptosis and senescence-progression induced tubular injury and loss of function, observed during diabetic nephropathy [8], [12], [13]. Kidneys with type 2 diabetic nephropathy display an accelerated senescent phenotype in defined renal cell types, mainly tubule cells, and to a lesser extent, podocytes [14]. A similar senescent pattern was observed when proximal tubule cell cultures where incubated under high-glucose media. Apoptotic cell death contributes to diabetic nephropathy (DN) and is present in acute (10 min) exposure of human proximal tubule epithelial cells (hPTEC) to high glucose (25 mM) [15], [16], [17], [18], [19], [20], [21]. High glucose induces a time-dependent dual effect consisting of an early proliferation and a late apoptosis (reminiscent of “crisis” post-proliferative senescence) [22].

Senescence is a tumor suppression mechanism blocking cell-proliferation that is induced by several stimuli, including oncogenic signaling and telomere shortening [23], [24], [25]. Experimental evidence supports that senescence involves DNA damage, increased ROS generation, accumulation of the cyclin-dependent kinase inhibitor p16INK4a, and/or p53 pathway activation [26], [27]. Senescence is a sensitizing pathway towards apoptosis and nephron loss. Indeed, if replicative senescence is bypassed, cells enter M2 or “crisis” and result in apoptosis [28], [29], [30].

Preliminary data suggested that the vitamin D pathway is significantly altered during diabetic nephropathy. Particularly, *CYP24A1* expression is greatly increased; a state that might lead to alternative gene expression and pathway activation. Indeed, analysis of the key players in the metabolism of vitamin D during diabetic nephropathy progression shows that CYP24A1 expression is significantly increased in the proximal tubules of db/db (C57BL/6 *Lpr −/−)* mice versus db/m+ (C57BL/6 *Lpr −/m+)* mice, as well as in animals fed a high fat diet for 8 weeks. We thus hypothesized that under normal conditions, circulating vitamin D levels are tightly controlled in the proximal tubules of the kidney through synthesis of the active metabolite 1,25(OH)2D3 (by CYP27B1) and through degradation (by CYP24A1) [31]. During type 2 diabetes progression, renal CYP24A1 is greatly elevated, impairing vitamin D metabolism and accelerating tubular injury through cellular senescence and apoptosis [32], [33]. Moreover *CYP24A1* promoter activity has been described to be induced by its own substrate, 1,25(OH)2D3, in a VDR dependant manner. In the present study, we investigated the mechanisms by which HG enhances CYP24A1 expression, and the consequences of this increased expression on the phenotype of tubular cells.

## Results

### CYP24A1 has a dramatically altered expression in kidneys of animals with type 2 diabetes

**Figure 1:**
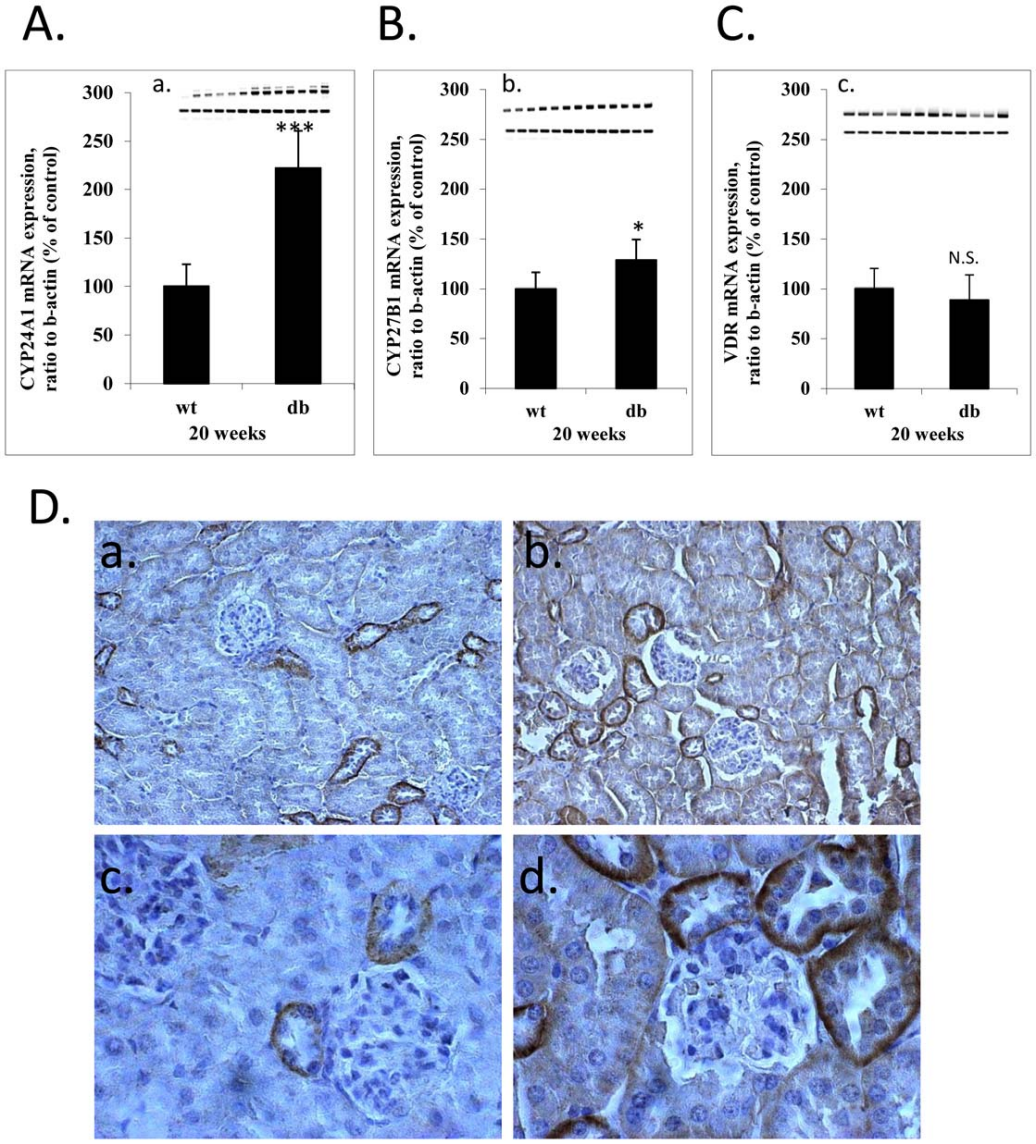
CYP24A1 is increased 2–3 fold in renal proximal tubules of diabetic mice. mRNA expression levels in renal proximal tubules of mice 20 weeks old were semi-quantified by RT-PCR/PCR, wild type compared to db/db (A) CYP24A1 (a) gel scan, (B) CYP27B1(b) gel scan, (C) VDR (c) gel scan. (*p<0,05; **p<0.01; ***p<0.001). (D) CYP24A1 immuno-staining of paraffin kidney sections (a,c) C57/BL6 wild type 24 weeks old, (b,d) C57/BL6-J *db/db* 24 weeks old (magnification X200 (a,b), X400 (c,d)).

In previous studies [16], [20], we have reported that multiple mRNA variations can be observed in the kidney proximal tubules between age-matched wild type C57BL6J male mice and *db/db*-C57BL6J male mice, performing GeneChip microarray. Data analysis indicated that vitamin D metabolism might be severely impaired, with an emphasis on the CYP24A1 catabolitic enzyme (data not shown). We confirmed our microarray results through analysis of a significant number of animals from a second similar study (Fig. 1A and 1D). Interestingly, as reported by other groups, *Cyp27b1* was slightly increased (Fig.1B and 1b), the major variation was found in *Cyp24a1* expression. Indeed, it was consistently and significantly increased in kidney proximal tubules from diabetic animals (Fig. 1A, 1a, and 1D).

We also looked at VDR expression, as its crucial role has been extensively described in scientific literature, but we found no real consistency in its variation (Fig. 1C and 1c). We suspect that aging and disease progression might have antagonistic signalling. Moreover, VDR expression is highly dependent on vitamin D metabolite concentrations. These concentrations can be altered by CYP24A1, leading to variable levels of VDR expression between individual animals. In the light of these results, we hypothesized that increased CYP24A1 radically changes local active vitamin D bioavailability in the renal proximal tubules, possibly leading to cellular instability and phenotypic changes. We looked further, as a result, into the specific role of vitamin D metabolism in kidney proximal tubules during diabetic nephropathy progression.

### CYP24A1 up-regulation in high glucose conditions is H_2_O_2_ -dependent

**Figure 2:**
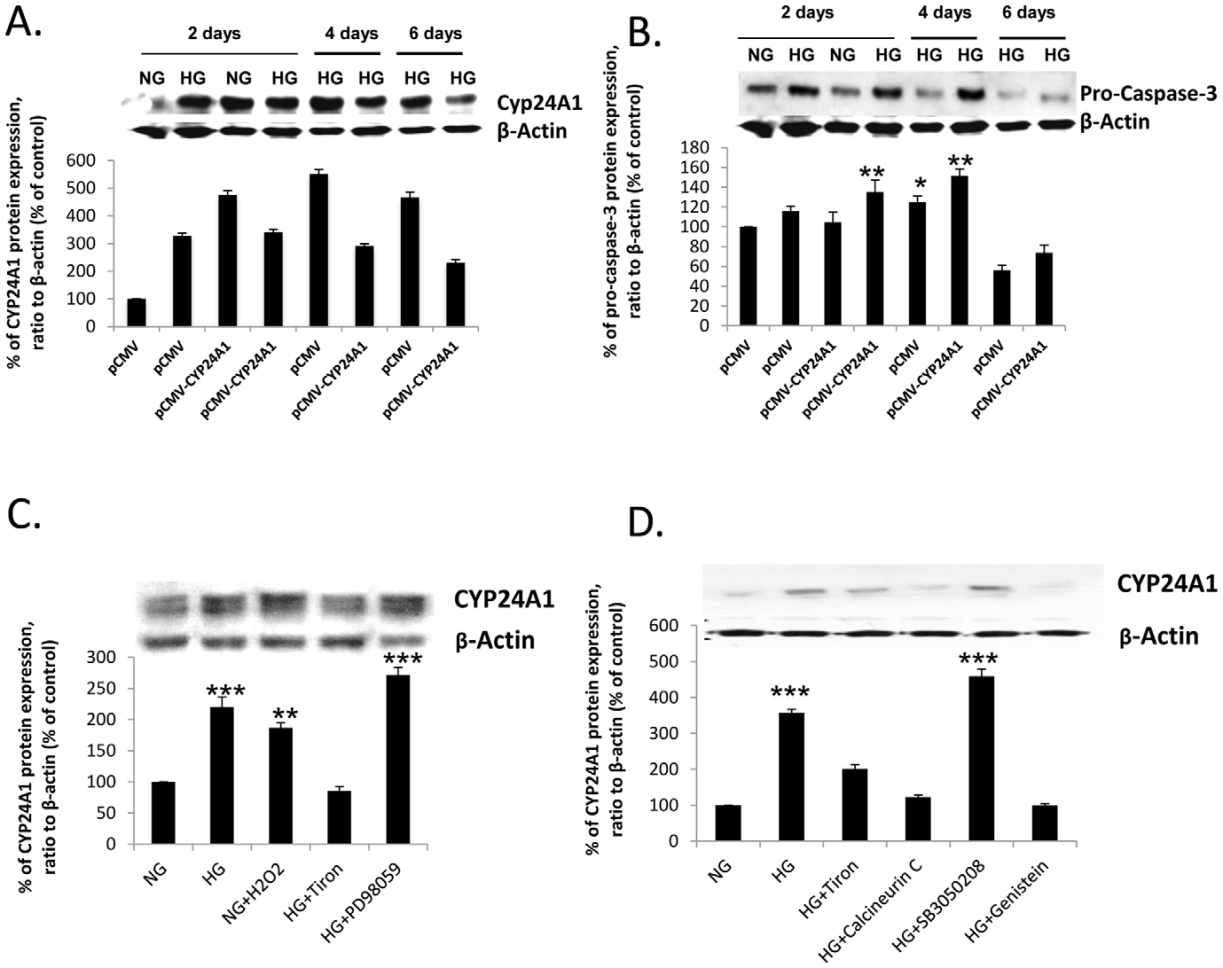
CYP24A1 increase in high glucose conditions is reactive oxygen species production and Protein kinase C activation-dependent. (A) Time course of CYP24A1 protein expression in 25 mM D-Glucose , transfected or not with pCMV-CYP24A1, (B) (A) Time course of pro-caspase-3 protein expression in 25 mM D-Glucose , transfected or not with pCMV-CYP24A1, (C) CYP24A1 protein expression levels in hPRPTCs cultured for 4 days with H_2_O_2_ or with 25 mM D-Glucose plus Tiron. (D) CYP24A1 protein variation in hPRPTCs cultured for 4 days with 25 mM D-Glucose plus PD98059, SB3050208, Calcineurin C or Genistein. (*p<0.05; **p<0.01; ***p<0.001)

To unravel the steps that lead to CYP24A1 up-regulation in diabetic conditions, we had to first verify that high glucose (25 mM D-Glucose, HG) could indeed induce a higher expression of CYP24A1. In a time course experiment (Fig.2A), we determined that a minimum of 2 days were necessary to see a significant increase in CYP24A1 protein expression (Fig.2A, C and D) in high glucose. VDR was also significantly increased, while CYP27B1 decreased initially (5 days) and showed a remarkable cell adaptation/regulation in the long term (10 days) (data not shown). These findings explain in part the results seen in older mice with diabetes: significant increase in CYP24A1, variable expression of VDR and a slight non-significant increase in CYP27B1. Furthermore, increased expression of CYP24A1 was parallel with an increased expression of pro-caspase-3 (Fig.2B), an important finding discussed in the second part of the paper as a possible explanation for increased apoptosis. It is important to note that our control conditions compared 25 mM D-Glucose (HG) to 5 mM D-Glucose with 20 mM D-Mannitol (NG) to avoid any bias due to difference of osmolarity.

Moreover, we suspected that the rise of reactive oxygen species was implicated in CYP24A1 increased expression. Thus, as shown in figure 2,C and D, we treated cells with H_2_O_2_ (10^−2^ M) or with 25 mM D-Glucose plus Tiron (10^−5^ M), a ROS scavenger, for 4 days, and looked at protein expression of CYP24A1. As expected, H_2_O_2_ could induce a significant rise in protein expression, while Tiron could inhibit the high glucose dependant CYP24A1 increased expression; thus, showing that this increase was ROS-dependant. Furthermore, we used inhibitors of three major possible molecular cascades possibly involved: PD98059 for ERK1/2, SB3050208 for p38 MAPK, Calcineurin C for PKC and Genistein at 10^−5^ M as a control inhibitor of CYP24A1 expression (Fig. 2C and D). Our results showed that p38 MAPK wasn't involved, nor was ERK 1/2. Only, PKC inhibition prevented CYP24A1 protein rise in high glucose conditions, probably through its control of VDR expression. We thus concluded that high glucose conditions can induce CYP24A1 expression in hPRPTCs via H_2_O_2_ activation of the PKC pathway.

### CYP24A1 expression regulates primary proximal tubular cell cycle profile status

**Figure 3:**
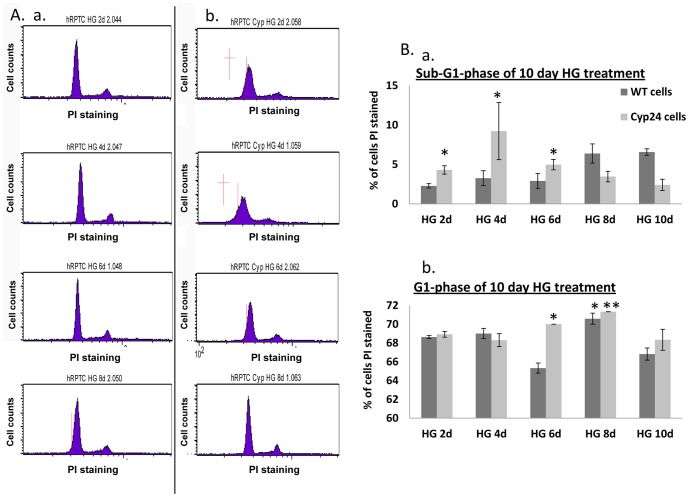
High Glucose and CYP24A1 over-expression induce G1 arrest by themselves, but synergistically induce apoptosis in hPRPTCs. (A) FACS cell cycle profiles of propidium iodide stained cells over a 8 day time course in high glucose, (a) hPRPTCs, (b) hPRPTCs transfected with pCMV-CYP24A1. (B) % of cells in a specific cell cycle phase over 10 days time course in high glucose, (a) SubG1, (b) G1. (*p<0.05; **p<0.01; ***p<0.001)

**Figure 4:**
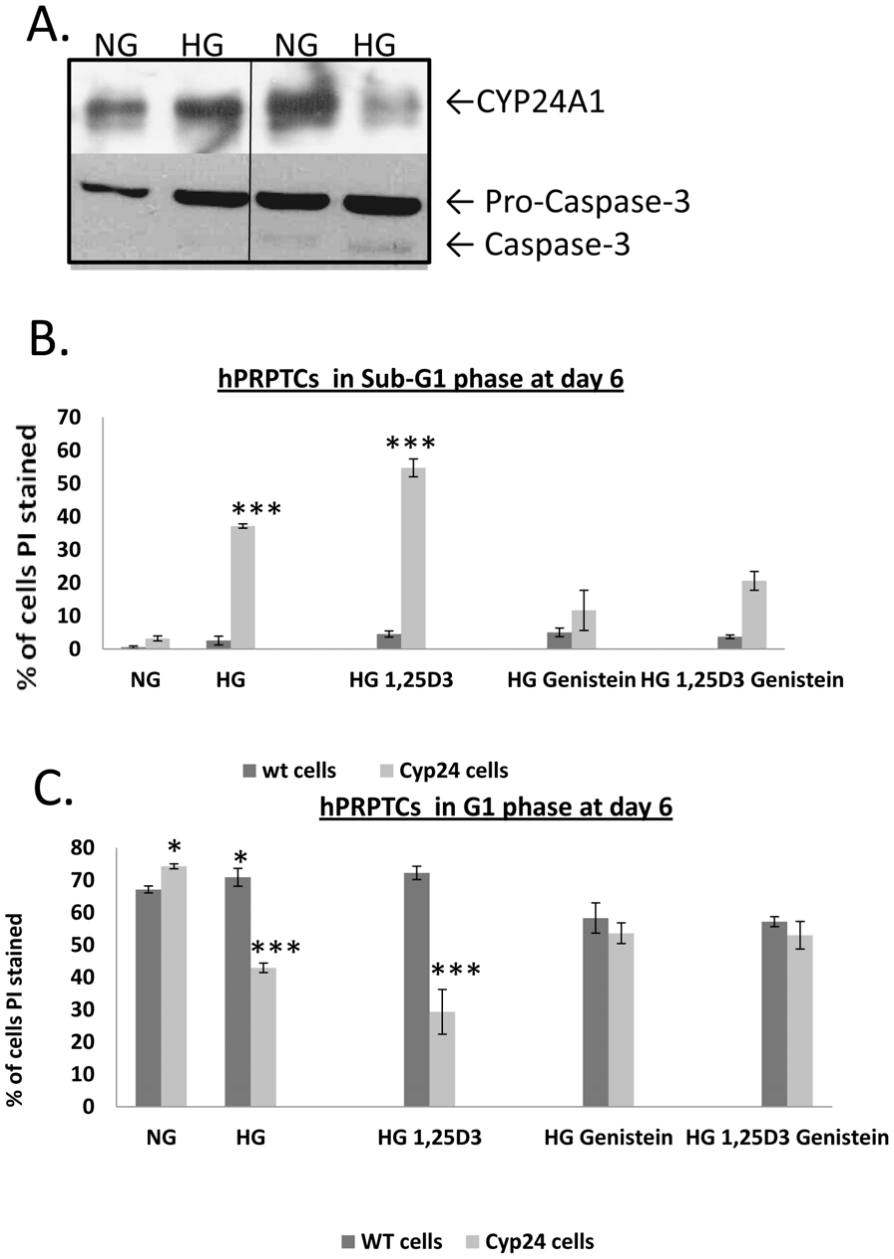
G1 arrest-induction in high glucose of hPRPTCs is partly CYP24A1-dependent, but apoptosis induction is entirely CYP24A1-dependent. CYP24A1 expression and Caspase-3 activation profiles in hPRPTCs cultured for 4 days under NG or HG and transfected or not with pCMV-*CYP24A1* (A). hPRPTCs in NG, hPRPTCs in HG, hPRPTC in HG with 10^−9^ M Calcitriol, hPRPTCs transfected with pCMV-*CYP24A1* in NG, hPRPTCs transfected with pCMV-*CYP24A1* in HG, and hPRPTCs transfected with pCMV-CYP24A1 in HG with 10–6 M Genistein. % of cells in SubG1after 6 days incubation (B), and G1 (C). (*p<0.01; **p<0.05; ***p<0.001)

In order to understand the role of CYP24A1 in primary proximal tubular cells, we performed FACScan analysis of the cell cycle profile changes of native cells compared with cells transfected with pCMV-*CYP24A1*. We then compared naive cells with cells transfected with pCMV-*CYP24A1* and incubated them with increasing periods of time (2, 4, 6, 8days) in 25 mM D-Glucose. Results showed that CYP24A1 overexpression blocked cells in G1, while overexpression of CYP24A1 in 25 mM D-Glucose pushed cells into SubG1 (day 2) (Fig. 3A,a and b, and 4B and C). Cells that do not die are found blocked in G1. Naive cells incubated in 25 mM D-Glucose enter G1 arrest after longer periods of incubation (day 6), but showed a small subG1 fraction after 8 days of high glucose incubation (Fig.3). In another set of experiments, we challenged native cells and cells overexpressing CYP24A1 with high glucose and either a CYP24A1 transcription inhibitor (Genistein 10 µM) or a CYP24A1 substrate and inducer (1, 25 D3 called Calcitriol 10 nM) (Fig.4). Results show again that CYP24A1 overexpression blocked cells in G1, while overexpression of CYP24a1 in 25 mM D-Glucose pushed cells into SubG1. 1, 25D3 could increase SubG1, while Genistein could block G1 arrest (Fig. 4B, and C).

Also, we could detect increased pro-caspase 3 expression and more importantly, activation of caspase-3, when cells overexpressing CYP24A1 were incubated in 25 mM D-Glucose for 4 days (Fig.4A). In light of these data, we hypothesized that increased CYP24A1 expression observed in high glucose conditions might play a role in cellular senescence and apoptosis.

### CYP24A1 increased expression is responsible for G1 arrest and apoptosis induction in high glucose

In order to determine whether the observed phenotype was actually due to CYP24A1 increased expression, as well as to discount the possibility that we witnessed two concomitant non-related phenomena, we studied high glucose effects in suppressed CYP24a1 conditions. To do so, we used siRNA against CYP24A1 and siRNA against VDR to ascertain 1, 25D3-dependency.

**Figure 5:**
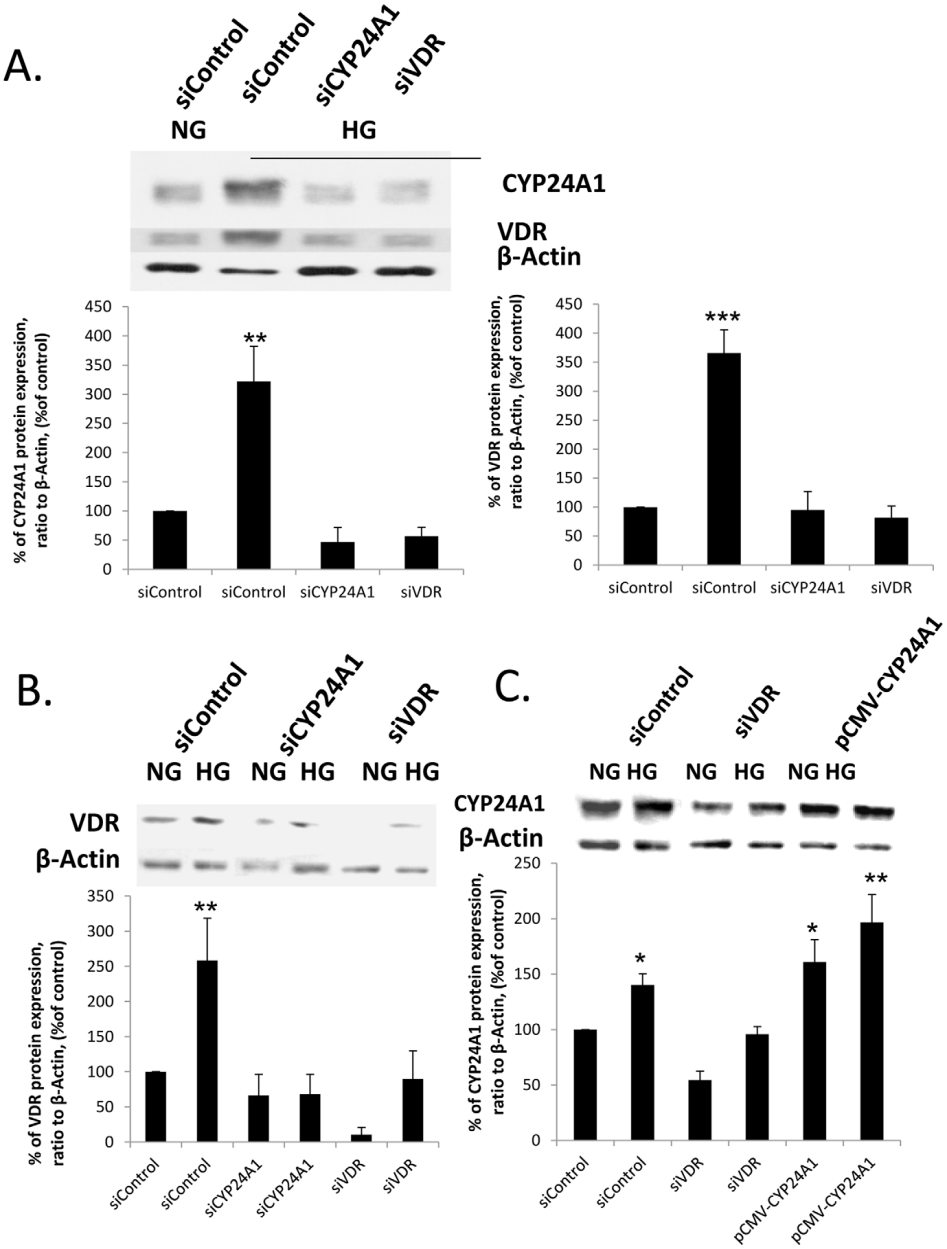
VDR expression is CYP24A1-dependent and *vice-versa* CYP24A1 expression is VDR dependent. (A, B and C) CYP24A1 and VDR protein expression analysis by western blotting 4 days after transfection with siRNAs scrambled (control), si*CYP24A1*, si*VDR* or pCMV-*CYP24A1*. (*p<0.05; **p<0.01; ***p<0.001)

**Figure 6:**
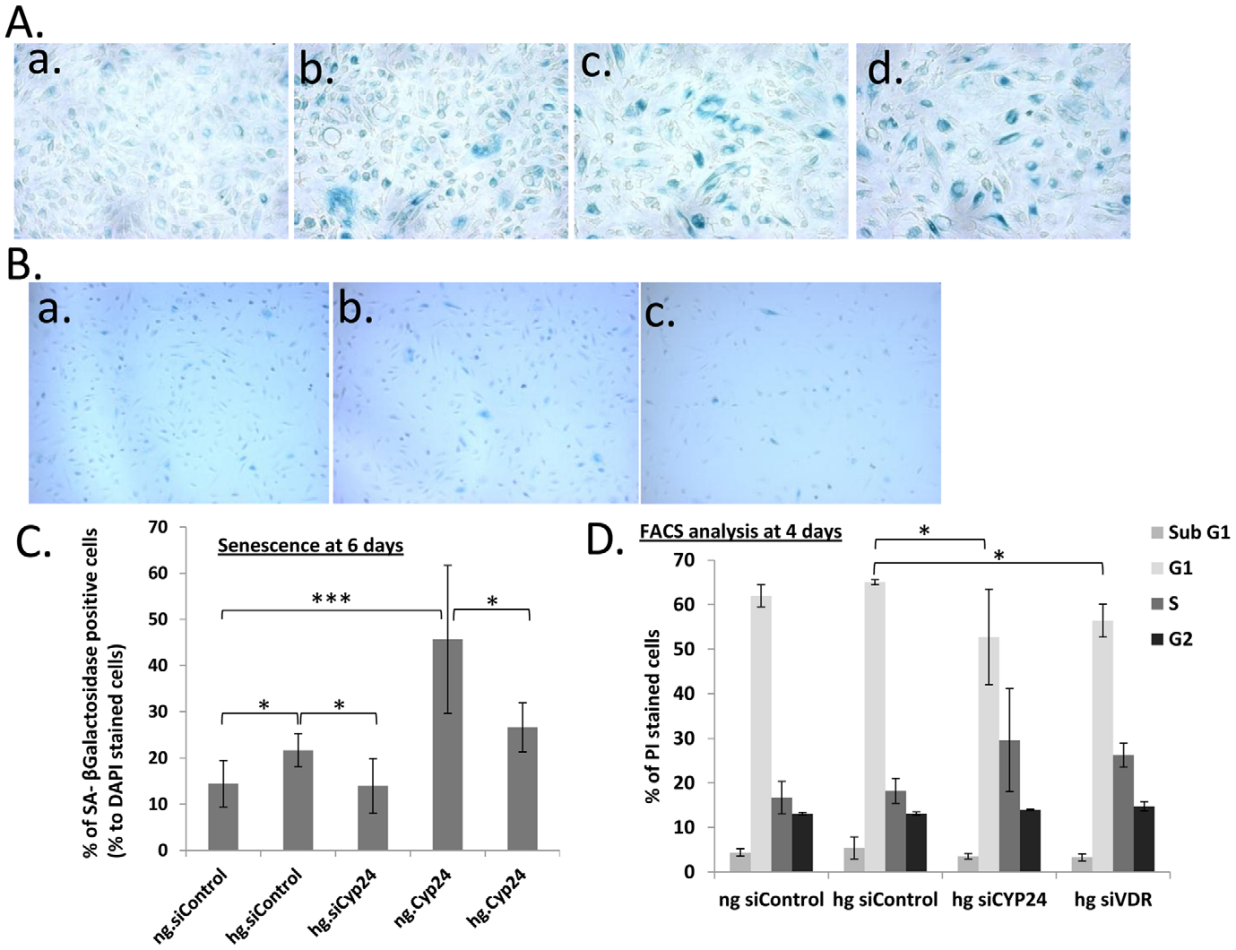
High Glucose G1 arrest and senescence induction is CYP24A1-dependent. (A) Senescence analysis by SA-βGalactosidase assay after 6 days (a) hPRPTCs in NG, (b) hPRPTCs in HG, (c) hPRPTC after transfection with pCMV-*CYP24A1* in NG (d) hPRPTC after transfection with pCMV-*CYP24A1* in HG. (magnification X400). (B) Senescence analysis by SA-βGalactosidase assay after 6 days hPRPTCs after transfection with siRNAs (a) scrambled (control) in NG (b) scrambled (control) in HG and (c) si*CYP24A1* in HG. (magnification X200). (C) % of senescent cells after 6 days incubation. (D) FACS cell cycle profiles of propidium iodide cell-stained, % of cells in a specific cell cycle phase after 4 days incubation in HG. (*p<0.05; **p<0.01; ***p<0.001)

Both siRNA were transfected by electroporation and were effective in suppressing their respective protein expression in high glucose conditions (Fig. 5A). We also determined that in high glucose conditions, there was a slight increase in CYP24A1 expression in the presence of siRNA for VDR, suggesting that high glucose could also partially increase CYP24A1 independently of VDR-substrate promoter activation. Surprisingly, in high glucose, repressing CYP24A1 expression prevented VDR increase, suggesting a positive feed-back loop between CYP24A1 and VDR (Fig.5A B and C). Furthermore, when we analyzed the effect of siRNA transfection in hPRPTC in high glucose conditions, we could see that G1 arrest and apoptosis induction in high glucose conditions was completely dependent on CYP24a1. Indeed, transfection of CYP24A1 siRNA increased basal cell viability (decreased sub-G1 pic and G1 arrest). In contrast, CYP24A1 overexpression induced senescence (increased G1 arrest) in normal conditions and induced apoptosis (increased sub-G1 fraction) in high glucose conditions (Fig. 6D).

### CYP24A1 activity induces p16-independent senescence and promotes apoptosis in high glucose

To confirm that the increase in G1 is indeed cellular senescence, we used SA-β-galactosidase assay to measure senescence-dependant gene activation. We did in fact see increased cellular senescence in cells overexpressing CYP24A1 that exceeded cellular senescence induced by high glucose alone, as shown by increased numbers of blue-stained cells (Fig. 6A, a,b,c, and d, and 6C). No cellular senescence was observed in cells incubated in high glucose but transfected with si*CYP24A1* (Fig. 6B,a,b and c, and 6C).

### Control of CYP24A1 increase protects animals on high fat diet from weight gain and hyperglycemia

Three different groups of mice: Wild type C57/BL6 male mice, *CYP24+/−* C57/BL6 male mice and *CYP24 −/−* C57/BL6 male mice were fed a high fat diet (60% fat) for 8 weeks, starting at 8 weeks of age. After 8 weeks, mice were weighted, sacrificed after blood sample collection by intra-cardiac puncture, and we collected the kidneys. Kidneys were prepared for analysis as follows: half a kidney cut sagitally was be fixed in PBS 10% formaldehyde and stored in paraffin blocks or frozen. One and a half kidney was used to isolate the proximal tubules. Blood samples were used to measure the 1, 25 (OH)2 D3 circulating levels.

**Figure 7:**
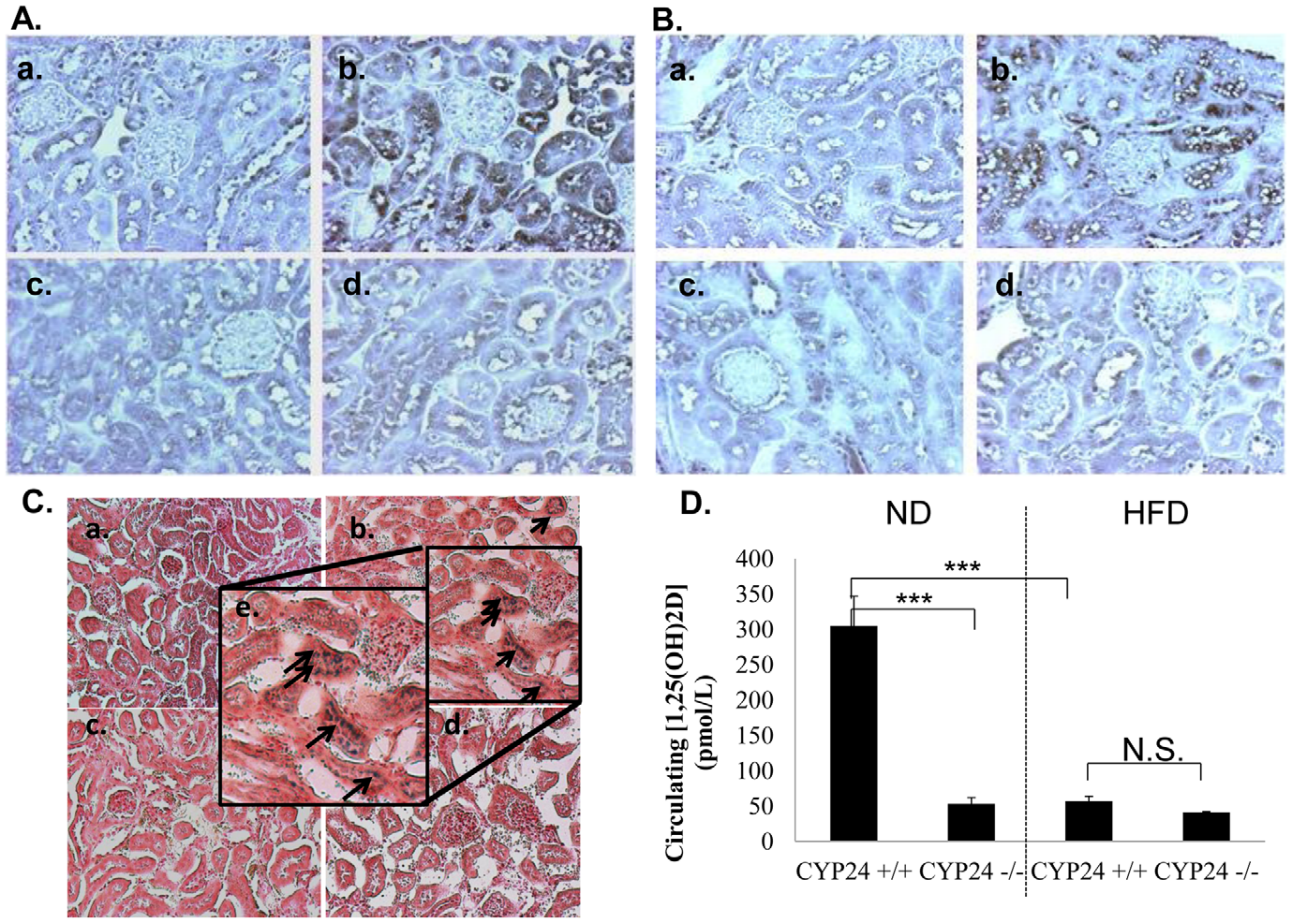
Control of CYP24A1 high fat fed animals prevents increase of pro-caspase-3 protein expression. (A) Cyp24A1 immuno-staining, proteins (a) C57/BL6 wild type on normo-diet, (b) C57/BL6 wild type on high fat diet, (**c**) C57/BL6-*Cyp24A1 −/−* on normo-diet, (d) C57/BL6-*Cyp24A1 −/−* on high fat diet. (magnification X400). (B) Caspase-3 immuno-staining, proteins (a) C57/BL6 wild type on normo-diet, (b) C57/BL6 wild type on high fat diet, (**c**) C57/BL6-*Cyp24A1 −/−* on normo-diet, (d) C57/BL6-*Cyp24A1 −/−* on high fat diet. (magnification X400). (C) SA-βGalactosidase activity appears in tubular structures of high fat fed animals, but not in C57/BL6-*Cyp24a1 −/−* animals, as shown by the blue staining. (a) C57/BL6 wild type on normo-diet, (b and e) C57/BL6 wild type on high fat diet, (c) C57/BL6-*Cyp24a1 −/−* on normo-diet, (d) C57/BL6-*Cyp24a1 −/−* on high fat diet. (magnification X200 (a,b,c,d) and X400 (e)), (D) Circulating levels of 1,25(OH)2D3 in C57/BL6 wild type and C57/BL6-*Cyp24a1 −/−* , on either normo or high fat diet, at day of sacrifice (16 weeks) . (Each group with N = 4 animals and *p<0.05; **p<0.01; ***p<0.001)

Our results clearly showed that 8 weeks on a high fat diet induced a significant increase in CYP24A1 expression and pro-caspase-3 expression that is concentrated in proximal tubules. Furthermore, when kidney sections were immuno-stained with anti-CYP24A1 (Fig. 7A) or anti-pro-caspase-3 antibodies (Fig. 7B), we could see no increased staining in *Cyp24A1 −/−* animals high fat fed kidneys, in contrast to wild type animals on high fat diet. This shows that pro-caspase-3 expression might be CYP24A1 activity dependant. We further analyzed tubular senescence in kidney from animals on high fat diet using the SA- βGalacosidase activity assay. We could clearly detect senescent tubular cells in kidneys from wild type animals on high fat diet, as shown by the blue stained cells (Fig. 7Cb and e). No blue cells were detectable in the *Cyp24A1 −/−* animals high fat fed kidneys (Fig. 7Cd). The blood samples showed us that contrary to the LepR −/− model, where 1, 25 (OH)2 D3 is unexpectedly increased [34], in the high fat model we can see a clear reduction in circulating levels of 1, 25 (OH)2 D3 of animals high fat fed (Fig.7D and supplementary data table S1). Moreover, as expected we saw that levels are further diminished in the CYP24 −/− mice.

**Figure 8:**
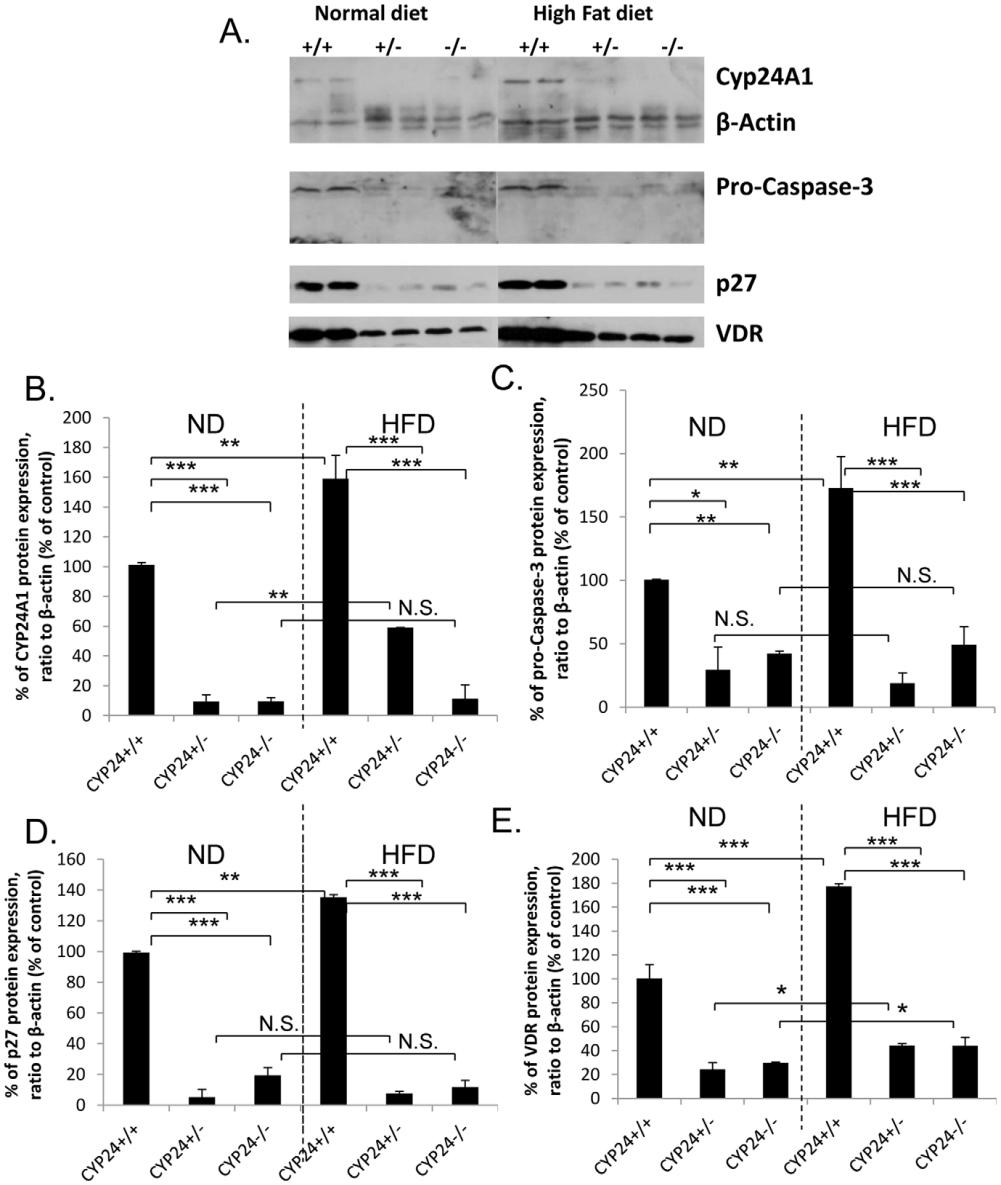
CYP24A1, pro-caspase-3, VDR and p27 rise in high fat fed animals, but not in C57/BL6-*Cyp24a1 −/−* animals high fat fed. (A) Rise and disappearance of Cyp24A1, pro-caspase-3, VDR and p27, in animals after 8 weeks of high fat diet. (B) semi-quantification of CYP24A1 protein expression, (C) semi-quantification of pro-caspase-3 protein expression, (D) semi-quantification of p27 protein expression, (E) semi-quantification of VDR protein expression. (Each group with N = 6–8 animals and *p<0.05; **p<0.01; ***p<0.001)

Also, in freshly isolated proximal tubules from these animals, we could see increase of CYP24A1, VDR, pro-apoptotic pro-caspase-3 and pro-senescence p27 protein levels in animals high fat fed (Fig.8). *Cyp24A1 −/−* animals high fat fed displayed no such increase, on the contrary, we obtained a near disappearance of pro-caspase-3 (Fig.8C) and p27 (Fig.8D), as well as a significant reduction of VDR (Fig.8E) protein expression suggesting that CYP24A1 activity might somehow control their expression (Fig.8B).

## Discussion

It is widely accepted that a normal vitamin D metabolism is required to maintain healthy body functions, and proper cellular differentiation. Many pathological disorders come from or lead to disturbed vitamin D metabolism. It seems that diabetic nephropathy is one of many. Nevertheless most of our knowledge usually derives from cancer or immortalized cell cultures rather than from *in vivo* studies or isolated primary cells. In this study we tried to bring a small milestone into this gap. Diabetes conditions clearly destabilize vitamin D metabolism, and our study suggests that increased CYP24A1 activity is a major contributor to this effect. Obviously patients not supplemented with vitamin D may be more likely to be deficient in 1, 25(OH)2 D3 as it will be catabolised as soon as it is produced by the kidney. It has also been shown that insulin sensitivity is 1, 25(OH)2 D3-dependant [35], [36], [37], [38]. This suggests that its decrease might accelerate diabetes onset.

At the same time, instead of gaining much needed 1, 25(OH)2 D3, supplemented patients may actually see a kidney specific increase in CYP24A1 specifically destabilizing Vitamin D metabolism in the kidney. Our data from diabetic mice kidneys certainly show a 2–3 fold increase in CYP24A1, specifically in the renal proximal tubules. Our results from human primary renal proximal tubules uncovered a crucial role of CYP24A1 in inducing senescence and promoting apoptosis in high glucose conditions. When CYP24A1 activity was blocked by siRNA expression or Genistein, apoptosis was also inhibited. This series of events strongly suggest that CYP24A1 activity is important for apoptosis induction. This might be tissue specific, as some studies have shown that inhibiting CYP24A1 might promote apoptosis instead [39]. Nevertheless, these studies were conducted in cancer cells, which could also explain the difference in cell response. It is possible that vitamin D metabolism would impact organ cells differently depending of there being in normal conditions, or pathologic conditions. It is known that CYP24A1 promoter induction is substrate dependant, which suggests that diabetic patients supplemented with vitamin D may have an even higher kidney expression of CYP24A1. We certainly see in our model that increase in CYP24A1 goes along with increased apoptosis in high glucose conditions. Mechanistically, it is explainable by the increase in pro-caspase-3 expression and activation in high glucose. It seems that pro-caspase-3 expression is CYP24A1 activity dependant. As our animals lacking CYP24A1 also totally lack pro-caspase-3, we think that pro-caspase-3 expression might be vitamin D dependent.

Moreover, it has been suggested for some time that VDR expression is also influenced by vitamin D, but no molecular mechanism has yet been defined to account for this observation. To our consternation, lack of CYP24A1 led to a significant decrease in VDR protein expression. Results from our siRNA experiments and our high fat study with CYP24A1 KO animals clearly show a near disappearance of VDR protein expression in renal proximal tubules and in hPRPTCs. This contradicts previous data on CYP24A1 KO mice that report an increase in VDR mRNA expression in the kidney of these animals [40]. This dichotomy might be due to either the fact that total kidney mRNA is different from isolated proximal tubules mRNA, or it might be a tendency of the cells to compensate for lack of protein expression. The later would suggest that VDR is regulated by vitamin D at the translational level. Also it opens the hypothesis that VDR regulation might be in a repressive fashion, where 1,25 D3 actually represses VDR expression, and its accelerated disappearance due to increase CYP24A1 would alleviate this repression. It would explain why in a context were CYP24A1 is not present, VDR would be highly repressed. The other possibility is that CYP24A1 activity is necessary for VDR expression. Nevertheless, this latest hypothesis is less likely as most tissues express VDR, but not necessarily CYP24A1 whose expression often needs to be induced.

In this context we can suggest a new hypothesis. Pro-Caspase-3 promoter is riddled with Sp-1 sites, and it has been shown in the literature that VDR can bind Sp-1 sites to activate gene translation. As an example, p27 expression has been shown to be VDR-Sp-1 binding dependent [41]. In our CYP24A1 KO animals there was also nearly no detectable p27. It is possible that pro-caspase-3 might be regulated in the same way, and that the absence of VDR expression in CYP24A1 KO mice would account for the lack of pro-caspase-3 expression.

In summary, it seems that CYP24A1 activity might be much more important than just catabolizing the 1,25 D3 metabolite. Also and not the least, our data suggest the possibility that vitamin D supplementation in diabetic patients might actually accelerate kidney injury through increased tubular apoptosis. This certainly raises a significant problem regarding patient supplementation. Indeed, more and more clinical data show that vitamin D has protective effects on outcome and mortality in diseases as varied as osteoporosis, cardio-vascular disease, aging etc. There might, however, be a parallel unforeseen adverse effect in particular cases. For example, from our data we predict that supplementing diabetic patients with vitamin D might accelerate tubular injury instead of slowing it, but we might achieve protection by simultaneously controlling CYP24A1 activity. Clearly, more studies will uncover the tissue specificity of vitamin D metabolism, and any possible disease tissue-specific differences.

## Materials and Methods

### Animal strains and High Fat Diet study

Animals for the studies were exclusively males and comprised: C57Bl/6 mice and homozygous *db/db* mice (B6.Cg+*Lepr^db/+^ Lepr^db/+^* on a C57BL/6J background) that were both purchased from Jackson Laboratories, (Bar Harbor, ME, USA) with a specific request for *db/db* mice to be with persistent hyperglycemia. The blood glucose levels on arrival were >25 mmol/l glucose and these levels were maintained throughout the experimental periods until 20 weeks of age. For the High Fat diet study, C57Bl/6 mice (from Jackson Laboratories) and CYP24A1-C57Bl/6 mice heterozygotes and KO [40] were fed either Chow (normo diet) or a 60% fat diet [42] from Bio-Serv, (Frenchtown, NJ, USA) for 8 weeks, starting at 8 weeks of age. The institutional ethics committee of the research center of Sacré-Coeur Hospital of Montréal in accordance with the CCAC Guidelines, specifically approved this study in protocol BREM03.

### Pathological analysis

Kidneys from mice were removed immediately after sacrifice. Formaldehyde (10%) fixed paraffin-embedded kidney sections of 5 µm were deparaffinized in xylene and rehydrated. Tissue sections (four to five specimens per group) were immuno-stained and analyzed visually under a light microscope by an observer unaware of the treatments. Immuno-histochemical examination was performed by the standard avidin-biotin-peroxidase complex method (ABC Staining System) (Santa Cruz Biotechnologies, Santa Cruz, CA, USA) as previously described. The sections were incubated with primary anti-CYP24A1 monoclonal antibody or Caspase-3 polyclonal antibody (both from Santa Cruz, USA) diluted 1∶50 for 16 h at 4°C; then, biotinylated secondary antibody was then added, followed by the addition of preformed ABC reagent supplied by kit. CYP24A1 and Caspase-3 were visualized by color development with 3, 3′–diaminobenzidine tetrahydrochloride. All sections were counterstained with hematoxylin, dehydrated, and covered with glass slips.

### Isolation of Mouse RPTs (mRPTs)

As soon as kidneys were removed, rinsed in ice-cold DMEM and decapsulated, the cortices were separated from the medulla. Proximal tubules were isolated by Percoll gradient as described previously [21]. Aliquots of freshly-isolated mRPTs from individual mice were immediately used for protein isolation.

### Cell culture and electroporation

Primary Renal Proximal Tubule Cells (Normal Human, from ATCC, Manassas, VA US), were cultured in Renal Epithelial Cell Basal Medium (ATCC) supplemented with Fetal Bovine Serum 0,5%, Triiodothyronine 10 nM, rh EGF 10 ng/ml, Hydrocortisone Hemisuccinate 100 ng/mL, rh Insulin 5 µg/ml, Epinephrine 1.0 µM, Transferrin 5 g/ml and L-Alanyl-L-Glutamine 2.4 mM, from Renal epithelial Cell Growth Kit components (ATCC). Original stock is passage 2, and cells are maintained up to passage 8. Electroporation was performed in culture medium with the Cloning Gun (Tritech Research, Inc., Los Angeles, CA, US), at the ratio of 1–2 µg DNA/500 000 cells.

### Cell Cycle Analysis

Human primary renal proximal tubular cells (hPRPTCs) were washed twice in phosphate-buffered saline (PBS) and fixed by resuspension in 1 ml of ice-cold 70% methanol in PBS. Cell suspensions were centrifuged, washed, and resuspended in 1 ml of Sodium Citrate solution with 50 µg/ml propidium iodide (PI, from Sigma-Aldrich Co, Canada), 0.05% RNase A, and incubated at 37°C in the dark for 30 min. The cells were then analyzed by flow cytometry on a FACScan (Becton Dickinson, San Francisco, CA) in FL2 channel.

### SA β-Gal Staining

We used the BioVision Senescence Detection Kit (BioVision Research, Mountain View, CA, US). Briefly, hPRPTCs were washed twice with PBS and fixed in 10% formaldehyde for 15 min at room temperature. Then, the cells were washed twice with PBS and incubated at 37°C (without CO_2_) with fresh senescence-associated β-galactosidase staining solution, containing 1 mg/ml 5-bromo-4-chloro-3-indolyl-β-D-galactopyranoside (X-gal), 40 mM citric acid/sodium phosphate, pH 6.0, 5 mM potassium ferricyanide, 150 mM NaCl, and 2 mM MgCl_2_. The color was developed for 12 h at 37°C. Photographs of the light microscopy images of the cells were obtained (20×).

### Radio Immuno-Assay measurement of circulating levels of 1, 25 (OH)2 D3

We use intra-cardiac puncture to collect blood from mice before sacrifice. Serum was separated by centrifugation at 4 C, ×3000 g. Then the samples were processed as previously described [40] with the RIA (ImmunoDiagnostic Systems Ltd., Boldon, UK) kit.

### Western Blot Analysis

Western blot analysis was performed according to standard method. The proteins in cell extracts were quantified by Bradford (Bio-Rad Laboratories, Hercules, CA, USA). Briefly, 50 to 100 µg proteins were heated at 95 C for 5 min, centrifuged at 12,000 g for 5 min and loaded on sodium dodecyl sulfate (SDS)-12% polyacrylamide gel. After transfer by semi-dry blotting onto a PVDF membrane (Bio-Rad), it was blocked in 5% non-fat milk powder and 0.05% Tween 20 in PBS. The membrane was first blotted with mouse anti-CYP24A1 (Abnova), rabbit anti-VDR (Santa Cruz), goat anti-CYP27B1 (Santa Cruz), mouse anti-p27 (Santa Cruz) or goat anti-caspase-3 (Santa Cruz), and then re-blotted with mouse anti-β-actin (Santa Cruz) monoclonal antibodies (all secondary antibodies were from Santa Cruz) and chemiluminescent developing reagent (Denville Scientific Inc.). The relative protein densities and β-actin bands were semi-quantified by ImageJ software (N.I.H version, USA).

### RT-PCR Assays for Gene Expression

Total RNA was used in RT-PCR to quantify the amount of Cyp24a1, Cyp27b1, and VDR expressed in mRPTs according to the manufacturer protocol for M-MLV reverse transcriptase, (Invitrogen Canada Inc. Burlington, ON, CA). β-actin-S-5′ TCC TGT GGC ATC CAC GAA ACT 3′, β-actin-AS-5′ AAG CAT TTG CGG TGG ACG ATG 3′, m CYP24A1 S-5′GTG CTG GGC TCT AGC GAA GAC 3′, m CYP24A1 AS-5′ CAG CTC CCG GCT AGG TAC CAG 3′, m VDR S-5′ GTA CAG GAT GCT AAG CTG GTT 3′, m VDR AS-5′ CAT GCT GTT CTC CGG CTG GAA 3′, m CYP27B1 S-5′AAA CTA TGT AAT TCC CCA AGA 3′, and m CYP27B1 AS-5′ TCT ATC TAC AAA CTG TAG ATT 3′.

### Statistical Analysis

Data are expressed as means±SE. Student *t*-test was initially used to analyze the statistical significance between experimental groups. Then, data were analyzed by one-way ANOVA and Bonferroni test. A value of P<0.05 was considered statistically significant.

## Supporting Information

Table S1 **Circulating levels of 1,25(OH)2D in C57/BL6 wild type and C57/BL6-** ***Cyp24a1 −/−*** **, on either normal or high fat diet, at day of sacrifice (16 weeks) as well in C57/BL6 LepR ^−/−^ (DB).** Groups are the same as in figure 7.
(DOC)
