# Supplementary material for: CYP24A1 Exacerbated Activity during Diabetes Contributes to Kidney Tubular Apoptosis via Caspase-3 Increased Expression and Activation
Source: PLoS One. 2012 Oct 31;7(10):e48652. doi: 10.1371/journal.pone.0048652 (PMC3485377; doi:10.1371/journal.pone.0048652)
Supplement: Table S1 — Circulating levels of 1,25(OH)2D in C57/BL6 wild type and C57/BL6- Cyp24a1 −/− , on either normal or high fat diet, at day of sacrifice (16 weeks) as well in C57/BL6 LepR −/− (DB). Groups are the same as in figure 7. (DOC) [file pone.0048652.s001.doc]

**Circulating 1,25(OH)2D levels**

| **Genotype** | **Diet** | **[1,25(OH)_2_D] (pmol/L) (Mean±SEM and (N=4))** |
| --- | --- | --- |
| Cyp24a1^+/+^;LepR^+/+^ | Normal | 305 ± 42 |
| Cyp24a1^+/+^; LepR ^-/-^ | Normal | > 416 |
| Cyp24a1^-/-^; LepR ^+/+^ | Normal | 53 ± 9 |
| Cyp24a1^+/+^; LepR^+/+^ | High Fat | 57 ± 7 |
| Cyp24a1^-/-^; LepR ^+/+^ | High Fat | 41 ± 1 |

Supplementary data
